# The valdoni technique for bowel anastomosis. A rare complication

**DOI:** 10.1016/j.amsu.2019.06.006

**Published:** 2019-06-11

**Authors:** F. Carannante, M. Caricato, V. Ripetti

**Affiliations:** Department of General Surgery, Università Campus Bio-Medico, Via Alvaro del Portillo 21, 00128, Rome, Italy

**Keywords:** Colon cancer, Right emicolectomy, Ileocolic anastomose, Valdoni technique, Surgery complications

## Abstract

**Background:**

Valdoni technique involves leaving the mucosa layer, between the two anastomosed bowel tract intact, providing for a subsequent breakage of the intestine. It is a technique that allows you to keep the operating field clean.

*Surgical technique and Case Report*: We describe the Valdoni technique. We also report a case of 75 years old man affected by an ascending colon cancer with no metastasis. The patient underwent right hemicolectomy. Making the anastomose, the surgeon did the Valdoni technique, with no intraoperative complications.

The postoperative course was characterized by an abdominal pain with swollen abdomen, no flatus and vomit. A computed tomography (CT) revealed a sub-stenosis of the anastomose. We decided to do an urgent colonoscopy, with a resection of the mucosa layer not totally opened, using a Needle-knife Precut. The post procedure course was uneventful. The patient was discharged three days later.

**Conclusion:**

Valdoni technique allows the surgeon to keep the operating field clean. It is a valid alternative when the surgeons have to make a colonic anastomosis, doing open surgery.

## Highlights

• Valdoni technique is a surgical procedure, which allows to keep the operating field clean, doing open surgery.
• When enterotomy is done, we open only the adventitia, muscolaris and sub-mucosa layers, leaving the mucosa layer intact.
• This type of anastomosis could also reduce hospital costs because the surgeon does not need to use mechanical staplers.
• Resection of the mucosa layer with a Needle-knife Precut, normally used for ERCP.

## 1 Introduction

Anastomotic leak is the most feared complication after colo-rectal surgery [1], from 3 to 36% [2]. During right emicolectomy, this type of complication is rare [3]. Another complication is the stenosis of the anastomose [4,5], in particular for anterior rectal resection surgery and in case of Diverticulitis [6] and Radiotherapy [3].

Valdoni technique is a particular surgical procedure, which allows surgeon to keep the operating field clean [8,9]. When the enterotomy is done, the surgeon opens only the adventitia, muscolaris and sub-mucosa layers, leaving the mucosa layer intact, providing for a subsequent breakage of the intestine.

Many years ago, when the laparoscopic surgery was only a dream, ones of the important Italian Surgeon, Professor Valdoni, thought up and did a new bowel anastomotic technique. Surgeons, in memory of Professor Valdoni, called this surgical technique by his name: “Anastomosi sec. Valdoni”.

In literature we don't find any article which explain or show this technique, but in Italian hospital many surgeons remember this type of anastomosis which their schoolmaster handed down. Now Surgeons are not usually to use the Valdoni technique for anastomose. Surely, this is due to new surgical techniques, such as laparoscopy and robotic surgery with the use of mechanical staplers [7], but the Valdoni technique could be a valid alternative when the surgeon needs to do a bowel anastomosis doing open surgery. This type of anastomosis could also reduce hospital costs because the surgeon does not need to use mechanical staplers.

We report a particular case of Valdoni technique for right colon cancer, which was complicated by the narrowing of the anastomosis and partial opening of mucosa layer.

This work has been reported in line with the SCARE criteria [12].

## 2 The Valdoni technique

**Fig. 1:**
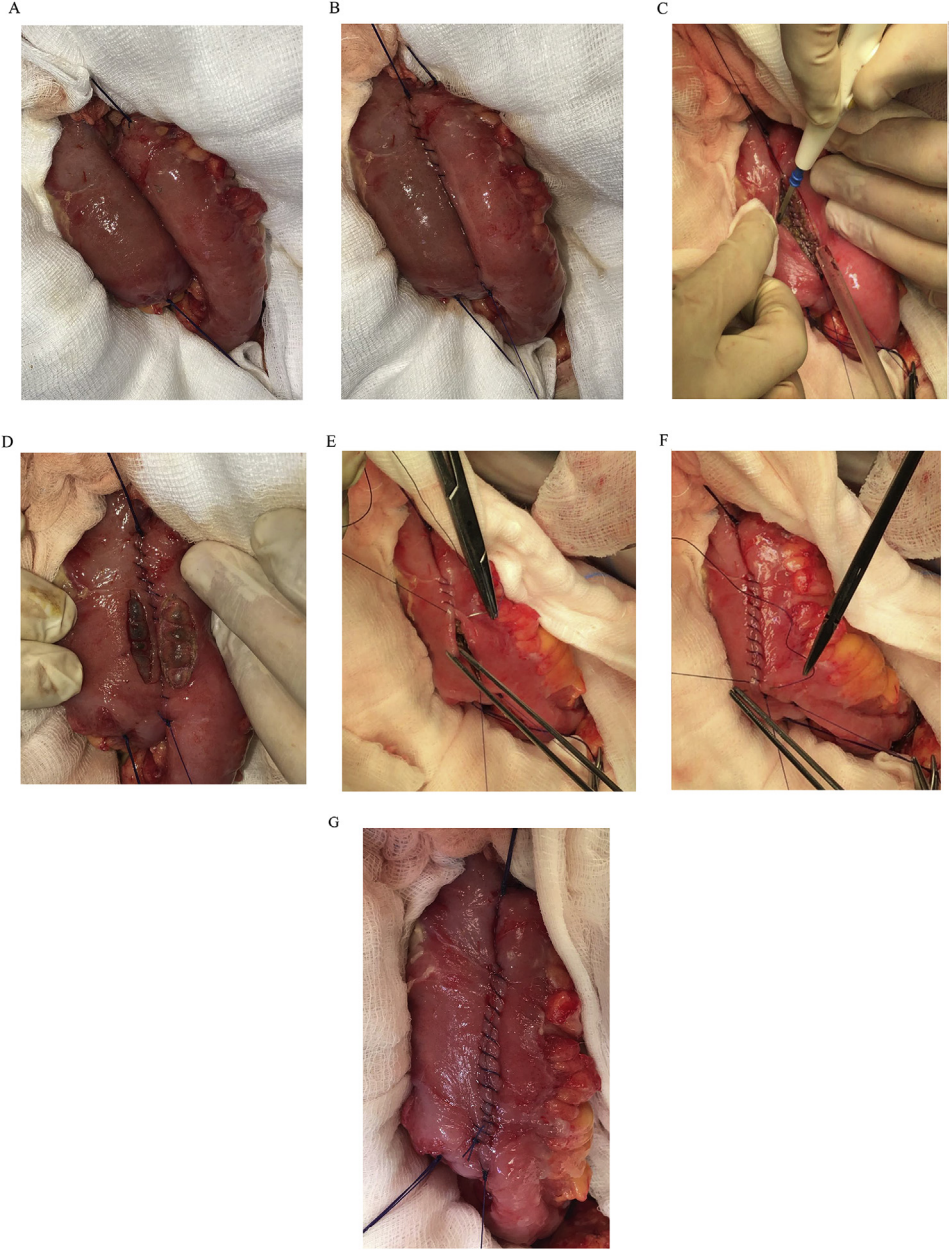

Suitable suture material for small bowel anastomosis, Vicryl (3/0), is used. At first, the two ends of the bowel are brought close together and corner sutures are done (Fig. 1 A). Using the corner sutures, the anastomosis is sewn continuously in a seromuscular fashion (Fig. 1 B).

When enterotomy is done, we open only the adventitia, muscolaris and sub-mucosa layers, leaving the mucosa layer intact, so we can avoid the faecal contamination, because the faeces cannot escape and dirty the operating field. (Fig. 1C–D).

Then, the anastomosis is sewn continuously making the intestinal continuity (Fig. 1EandF - G).

Only later, when the surgeon finished the anastomoses the mucosa layer could be broken, mechanically with finger pressure or later in the early postoperative days (the layer of the mucosa is the least vascularized layer).

## 3 Case report

A 75 years man was referred to our hospital for anaemia (Hb 11.6 g/dL), slimming and lack of appetite. He was a construction worker, not a smoker, from southern Italy. The patient had undergone a laparotomic appendectomy and laparotomic cholecystectomy, respectively 50 and 30 years ago. No other previous abdominal surgery was noted.

Total colonoscopy and computed tomography (CT) revealed ascending colon cancer with no distant metastasis. Tumour markers test results were within the normal range, with CEA of 3.35 ng/mL and CA19-9 of 11.6 U/mL.

We decided to do a laparoscopic right hemicolectomy. Unfortunately, due the previous surgeries a lot of adherences were found, so we decided to convert to open right hemicolectomy. When we have to make the anastomose, the surgeon did a particular surgical technique, called Valdoni technique. Prof. Valter Ripetti performed the procedure. No intraoperative complications occurred.

Pathological examination showed that the tumour was a low differentiated adenocarcinoma (G3) of the ascending colon, which reached the subserosal layer, with no lymph node metastasis (0/67). The pathological staging was T3N0M0 (TNM classification).

The postoperative course was characterized by a delayed canalization. At the fifth post-operative day, the patient showed an abdominal pain with swollen abdomen, with no flatus and vomit.

**Fig. 2:**
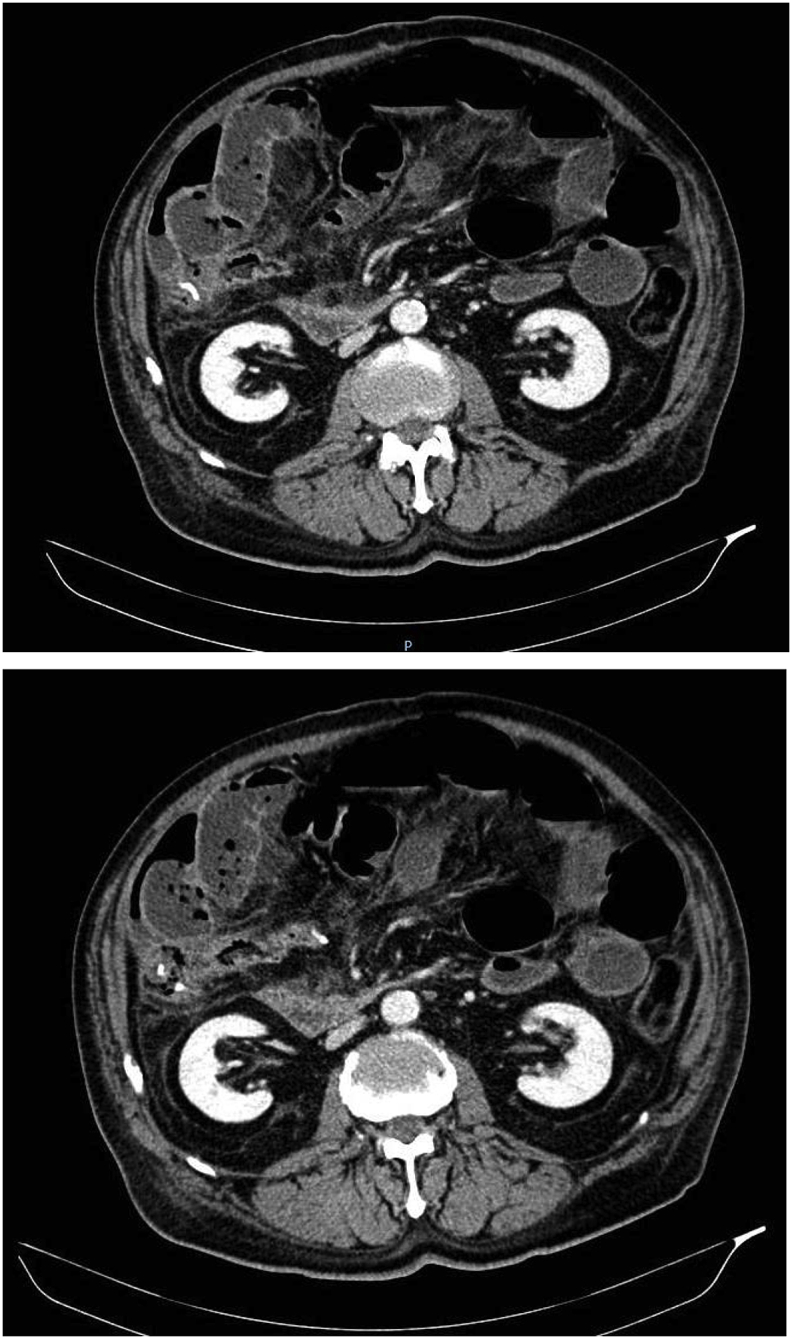
CT scans of the abdomen and pelvis, demonstrating the anstomostic stenosis.

A computed tomography (CT) revealed a sub-stenosis of the anastomose, with distension upstream of the intestinal loops (Fig. 1, Fig. 2B). No vascular problems were found; Superior and inferior mesenteric artery and vein were enabling the blood to flow properly.

**Fig. 3:**
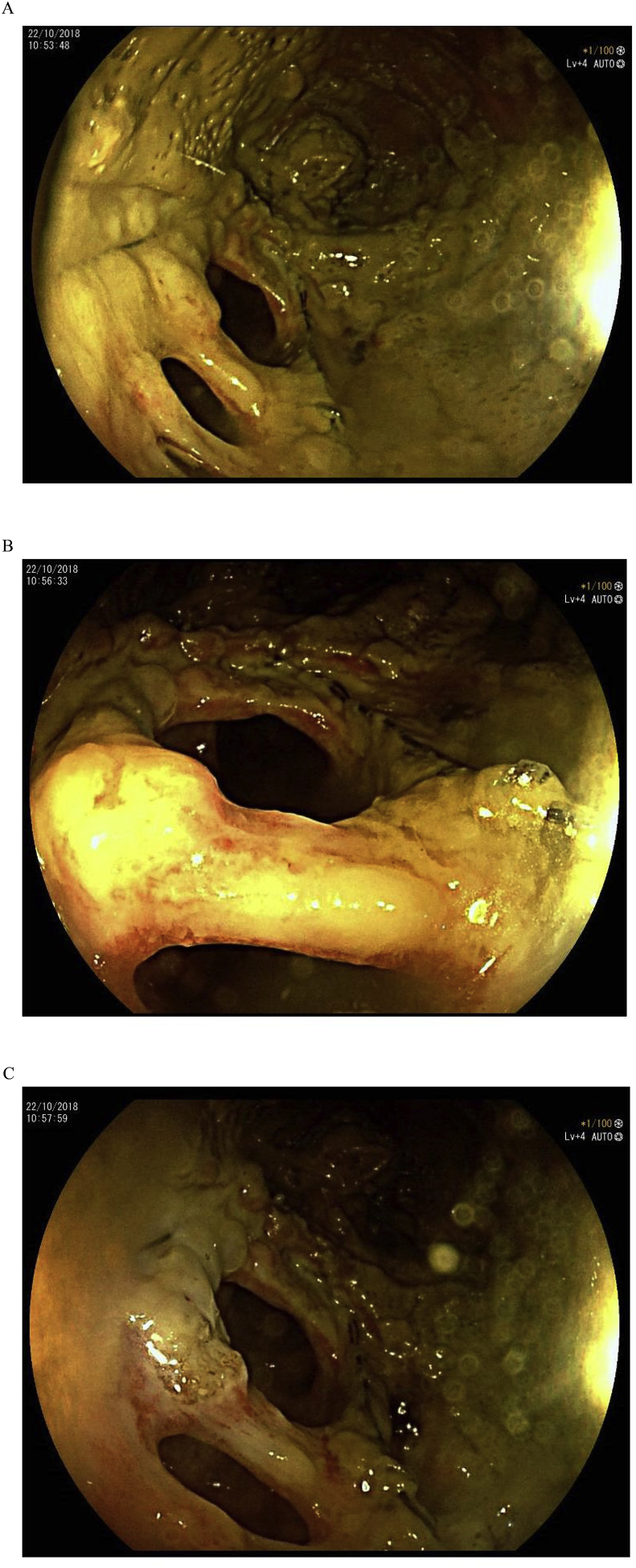
(A)Colonscopy image, demonstrating the partial rupture of the mucosa layer. (B)Colonscopy image, demonstrating the partial rupture of the mucosa layer. (C) Colonscopy image, demonstrating the partial rupture of the mucosa layer.

**Fig. 4:**
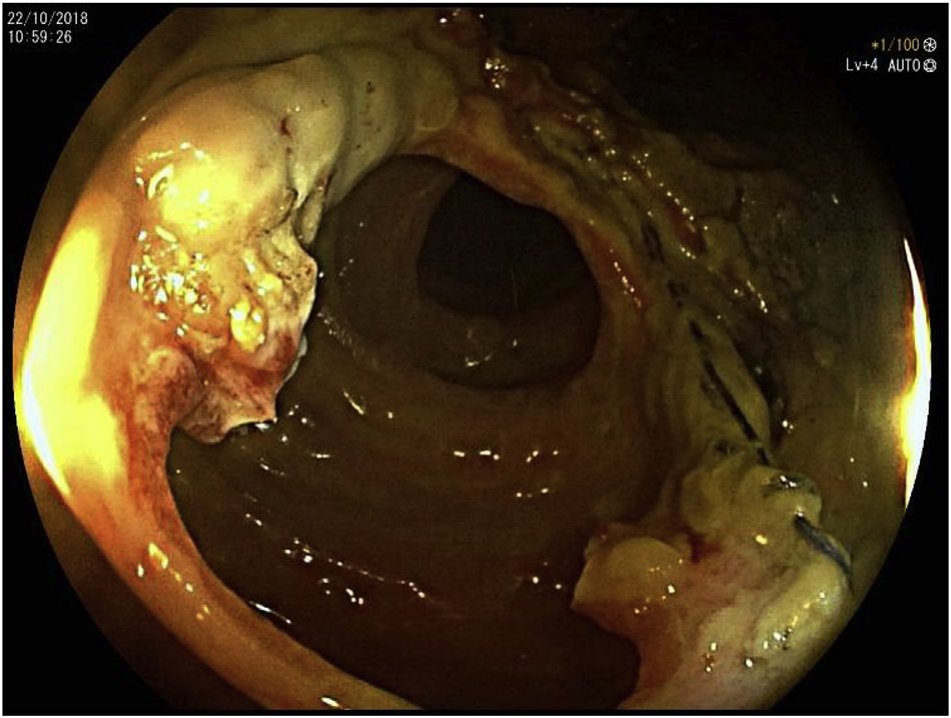
Colonscopy image, demonstrating the mucosa layer, after the endoscopy resection.

We decided to do an urgent colonoscopy, which showed a normal colonic mucosa. When the Endoscopist arrived at the end of the Colon, we found a narrowing of the anastomosis, with a partial opening of the mucosa layer (Fig. 3 A–3 B - 3C). At this point, we decided to do a resection of the mucosa layer with a Needle-knife Precut, normally used for ERCP (Endoscopic retrograde cholangiopancreatography) creating a large connection between ileus and colon (Fig. 4). We decided to use a Needle-knife Precut and not a Argon Plasma Coagulation (APC) instrument due to high risk of a colon perforation or explosion [10,11]. No complication occurred and the postprocedure course was uneventful. The patient was discharged after three days.

One month after, the CT scan showed no surgical complication and no distance metastasis.

## 4 Conclusion

Valdoni technique is a particular surgical procedure, which allows the surgeon to keep the operating field clean, doing open surgery. The partial opening of the mucosa layer is a complication not shown in the literature and, therefore, to be considered more than rare, but still to be taken in consideration. However, their resolution could be easy to solve.

To date, few surgeons use this technique, but it is a valid alternative when the surgeon have to make a colonic anastomosis, doing laparotomic surgery. Also, the reduction of health costs should be taken into consideration.

## Trial registry number

This study is not a first in man study and the registration in a publicly accessible database is not required.
